# Enzymatically polymerised polyphenols prepared from various precursors potentiate antigen-specific immune responses in both mucosal and systemic compartments in mice

**DOI:** 10.1371/journal.pone.0246422

**Published:** 2021-02-08

**Authors:** Rui Tada, Miki Ogasawara, Daisuke Yamanaka, Yasuhiro Sakurai, Yoichi Negishi, Hiroshi Kiyono, Naohito Ohno, Jun Kunisawa, Yukihiko Aramaki

**Affiliations:** 1 Department of Drug Delivery and Molecular Biopharmaceutics, School of Pharmacy, Tokyo University of Pharmacy and Life Sciences, Tokyo, Japan; 2 Laboratory for Immunopharmacology of Microbial Products, School of Pharmacy, Tokyo University of Pharmacy and Life Sciences, Tokyo, Japan; 3 Division of Mucosal Immunology and International Research and Development Center for Mucosal Vaccines, Department of Microbiology and Immunology, The Institute of Medical Science, The University of Tokyo, Tokyo, Japan; 4 Laboratory of Vaccine Materials and Laboratory of Gut Environmental System, National Institutes of Biomedical Innovation, Health and Nutrition (NIBIOHN), Osaka, Japan; Midwestern University, UNITED STATES

## Abstract

Despite significant modern medicine progress, having an infectious disease is a major risk factor for humans. Mucosal vaccination is now widely considered as the most promising strategy to defeat infectious diseases; however, only live-attenuated and inactivated mucosal vaccines are used in the clinical field. To date, no subunit mucosal vaccine was approved mainly because of the lack of safe and effective methodologies to either activate or initiate host mucosal immune responses. We have recently elucidated that intranasal administration of enzymatically polymerised caffeic acid potentiates antigen-specific mucosal and systemic antibody responses in mice. However, our earlier study has not confirmed whether these effects are specific to the polymer synthesised from caffeic acid. Here, we show that enzymatically polymerised polyphenols (EPPs) from various phenolic compounds possess mucosal adjuvant activities when administered nasally with an antigen to mice. Potentiation of antigen-specific immune responses by all EPPs tested in this study showed no clear difference among the precursors used. We found that intranasal administration of ovalbumin as the antigen, in combination with all enzymatically polymerised polyphenols used in this study, induced ovalbumin-specific mucosal IgA in the nasal cavity, bronchoalveolar lavage fluid, vaginal fluids, and systemic IgG, especially IgG1, in sera. Our results demonstrate that the mucosal adjuvant activities of polyphenols are not limited to polymerised caffeic acid but are broadly observable across the studied polyphenols. These properties of polyphenols may be advantageous for the development of safe and effective nasal vaccine systems to prevent and/or treat various infectious diseases.

## Introduction

Infectious diseases are a major risk factor in humans and are difficult to eradicate. Despite modern medicine progressing significantly, having an infectious disease is the second leading cause of death worldwide today [1,2]. Mucosal vaccination is now widely considered as the most promising strategy to counter infectious diseases caused by pathogens. Recently emerging mucosal vaccines are superior to conventional vaccines in terms of exerting protective immune responses not only in systemic compartments but also in mucosal surfaces, sites of invasion, and/or colonisation for most pathogenic microbes, such as *Streptococcus pneumoniae*, *Candida albicans*, and influenza virus [3–5]. On the other hand, conventional vaccines administered through parenteral injections (generally subcutaneous or intramuscular) are only capable of inducing antigen-specific immune responses in systemic compartments [6–9].

Despite the superiority of mucosal vaccines over conventional parental vaccines, only a few live-attenuated and inactivated mucosal vaccines have been approved for use in the clinical setting [10]. Both live-attenuated and inactivated mucosal vaccines contain whole pathogenic microbes. Hence, unexpected adverse effects due to the toxicity and antigenicity of the pathogens cannot be avoided. Therefore, the development of subunit mucosal vaccines that consist of a microbial antigen instead of whole microbes is required for the acceptance of mucosal vaccine systems in the clinical setting. However, no subunit mucosal vaccine has been approved, mainly because of the lack of safe and effective methodologies to either activate and/or initiate host mucosal immune responses by using mucosal adjuvants [11,12] or to deliver the microbial antigens to mucosal dendritic cells (mDCs) by means of antigen delivery vehicles [13,14]. This is because mucosal area has inherently poor capabilities to respond to antigenic proteins due to immune tolerance to the exogenous antigens [10,15–17]. Second, although mDCs located at the lamina propria are able to accomplish transepithelial antigen uptake, antigen transport into the lamina propria is crucial for initiating mucosal immune responses. However, macromolecules such as antigens are unable to cross a physical barrier composed of mucosal epithelial cells to access the mDCs located at the lamina propria; mucosal epithelial cells seal the entire mucosal tissue and form tight junctions blocking the paracellular route [18]. Numerous studies to overcome these problems have been reported in the literature. For instance, many microbe-derived mucosal adjuvants that trigger host innate immune responses via pattern recognition receptors, such as cholera toxin (CT), flagellin, and monophosphoryl lipid A, have been reported [19]. In addition, liposomes, nanogels, and bacterial components, which are utilised when some bacteria intrude the host tissues, have been reported to act as antigen delivery systems for mDCs [20–24]. However, such systems may cause side effects due to the presence of antigenic and toxic molecules largely derived from microbes. Hence, a safer and more effective system to promote antigen-specific immune responses in mucosal surfaces is needed for the successful development of mucosal vaccines.

Polyphenols are predominantly found in plants, and we ingest large amounts of polyphenols in our daily life [25]. We have long been focusing on these because of their immune-modulating effects on various immune cells. Polyphenols show anti-viral and anti-bacterial effects through their immune-enhancing characteristics [26,27]. We have found that orally administered polyphenols result in the activation of natural killer cells *in vivo* [28], and these polyphenols induce the production of cytokines from splenocytes *in vitro* [29–31]. Additionally, our previous studies investigating the cytotoxic and carcinogenic effects of the synthesised polymers revealed no cytotoxic or carcinogenic effects [28–31]. These findings clearly show that polyphenols positively activate innate immune systems without adverse effects in mice. In the course of our studies, we have recently uncovered that intranasal administration of enzymatically polymerised caffeic acid (pCA) potentiates antigen-specific mucosal and systemic antibody responses in mice by acting as a mucosal adjuvant. Specifically, intranasal administration of pCA, in combination with an antigenic protein, resulted in the induction of a higher titre of antigen-specific serum IgG1 and high levels of IL-4 and interferon-γ (IFN-γ) secretion in splenocytes following re-stimulation with an antigen *in vitro*. These findings suggest that pCA polarises a mixed Th1/Th2 type immune response [32].

However, our earlier study has not concluded whether such mucosal adjuvant effects are limited to the polymer synthesised from caffeic acid or are broadly observable in polymers synthesised from a range of phenolic compounds. In other words, the structure-activity relationship between the polyphenols synthesised from various precursors is yet to be elucidated. Thus, the goal of our study was to compare the effects of mucosal adjuvants from polyphenols from various precursors. We synthesised pCA, polymerised trans-ferulic acid (pFA), and polymerised trans-p-coumaric acid (pCoA) using horseradish peroxidase (HRP) as an enzymatic source, and then examined the mucosal adjuvant effects of various polyphenols using ovalbumin (OVA) as a model antigen by evaluating OVA-specific antibody production in both mucosal and systemic compartments in mice.

## Materials and methods

### Ethics statement

All animal experiments followed the guidelines of the Tokyo University of Pharmacy and Life Sciences. The institution’s committee for laboratory animal experiments approved each experimental protocol (P 17–26 and P 18–71).

### Materials

BALB/cCrSlc female mice (7 weeks old) were purchased from Japan SLC (Hamamatsu, Shizuoka, Japan) and were housed under specific pathogen-free conditions. All animal experimental protocols were approved by the Tokyo University of Pharmacy and Life Sciences committee for laboratory animal experiments (P17–26 and P18–71). 3-(3,4-Dihydroxyphenyl)-2-propenoic acid (commonly termed as caffeic acid; CA), *trans*-4-hydroxy-3-methoxycinnamic acid (commonly termed *trans*-ferulic acid; FA), and *trans*-4-hydroxycinnamic acid (commonly termed *trans*-*p*-coumaric acid; CoA) were all purchased from Tokyo Chemical Industry Co., Ltd. (Tokyo, Japan). HRP was obtained from Merck Millipore (Burlington, MA, USA). Low endotoxin (less than 1 EU/mg) egg white OVA and CT were acquired from FUJIFILM Wako Pure Chemical Corporation (Osaka, Japan).

### Preparation of the enzymatically polymerised polyphenols

**Fig 1:**
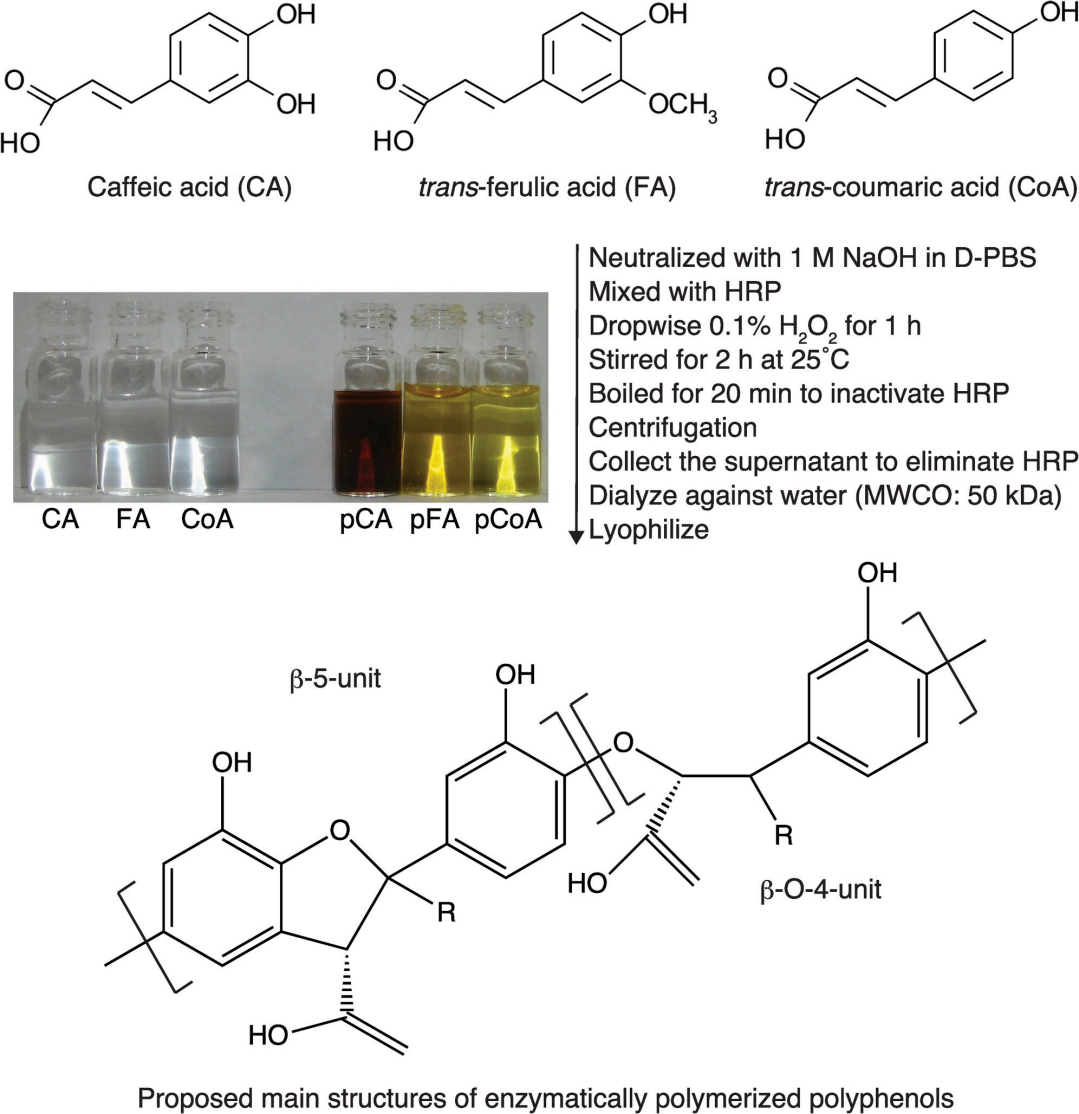
The scheme of synthesis, the structure of precursors, and the proposed major structure of enzymatically polymerised polyphenols used in this study. CA, caffeic acid; FA, *trans*-ferulic acid; CoA, *trans*-coumaric acid; pCA, polymerised caffeic acid; pFA, polymerised *trans*-ferulic acid; pCoA, polymerised *trans*-coumaric acid; HRP, horseradish peroxidase; MWCO, molecular weight cut-off; R, H or OH.

The lignin-like polymerised polyphenols were enzymatically synthesised with the enzyme HRP and the precursors CA, FA, or CoA, as reported earlier (Fig 1) [29]. Briefly, 200 mg of the various precursors were neutralised with 1 M NaOH and diluted to 10 mL with phosphate-buffered saline (PBS) containing 1 mg of HRP. 1.5 mol eq H_2_O_2_ was then added dropwise into a mixture of the precursor and HRP solutions while stirring at 25°C for 1 h. The reaction mixtures were stirred for another 2 h and subsequently heated for 20 min at 100°C to inactivate and precipitate the enzyme HRP. After centrifugation, the supernatant was collected and dialysed extensively using a dialysis membrane (MWCO 50,000) against deionised water for 2 days, and then lyophilised to obtain pCA, pFA, and pCoA. Endotoxin contaminants in this preparation were tested using the Endospecy ES-50M kit (Seikagaku Biobusiness Corporation; Tokyo, Japan) and indicated that the endotoxin content of pCA, pFA, and pCoA were low (231.5 pg/mg, 93.7 pg/mg, and not detected, respectively). All samples were dissolved in endotoxin-free PBS (FUJIFILM Wako Pure Chemical Corporation) as stock (10 mg/mL) and sterilised by filtration through 0.22 μm filter membranes (Osaka Chemical Co., Ltd., Osaka, Japan). The stock solutions were stored at -20°C in the dark until use. The immunomodulatory activities (cytokine secretion by splenocytes and T cell proliferation) of all polyphenols tested in this study did not change for at least one year under the storage conditions used.

### Immunisation and sampling schedule

Mice were divided into six groups and anaesthetised via intraperitoneal injection of 0.2 mL of a mixture containing 0.75 mg/kg of medetomidine, 4 mg/kg of midazolam, and 5 mg/kg of butorphanol tartrate. They were then immunised intranasally with the following: 1) PBS, 2) OVA alone (2.5 μg/mouse), 3) OVA (2.5 μg/mouse) plus pCA (100 μg/mouse), 4) OVA (2.5 μg/mouse) plus pFA (100 μg/mouse), 5) OVA (2.5 μg/mouse) plus pCoA (100 μg/mouse), or 6) OVA (2.5 μg/mouse) plus CT (1 μg/mouse). Each group of mice was immunised once weekly on days 0, 7, and 14. All samples were collected immediately after the mice were sacrificed via intraperitoneal injection of sodium pentobarbital (250 mg/kg; Tokyo Chemical Industry Co., Ltd., Tokyo, Japan). Blood samples were collected on day 21. The blood samples were allowed to clot at 25°C for 30 min, followed by incubation at 4°C for 60 min. The serum was then separated by centrifugation at 1200 × *g* for 30 min. Nasal wash fluid, bronchoalveolar lavage fluid (BALF), and vaginal wash fluid were collected in 200 μL, 1 mL, and 100 μL of cold PBS, respectively [20,33]. All samples were stored at –80°C until enzyme-linked immunosorbent assay (ELISA) analysis.

### ELISA for evaluating antigen-specific antibody titer

To evaluate the induction of antigen-specific IgA in the nasal and vaginal wash, BALF, and antigen-specific IgG in serum and BALF, a 96-well Nunc MaxiSorp plate (Thermo Scientific, Waltham, MA, USA) was coated with 1.25 μg of OVA dissolved in 0.1 M carbonate buffer (pH 9.5) and incubated overnight at 4°C. The plate was then washed with PBS containing 0.05% Tween 20 (PBST) and blocked with 1% bovine serum albumin (BSA; Wako Pure Chemical Corporation) containing PBST (BPBST) at 37°C for 60 min. The plate was washed and incubated with samples for 60 min at 37°C. Plates were washed with PBST, treated with peroxidase-conjugated anti-mouse IgA, IgG, IgG1, IgG2a, or IgG2b secondary antibody (SouthernBiotech; Alabama, USA) in BPBST, and developed using a tetramethylbenzidine (TMB) substrate system (KPL, Maryland, USA). Colour development was terminated using 1 N phosphoric acid and the optical density was measured at 450 nm (reference filter 650 nm) using a Synergy HTX Multi-Mode Microplate Reader (BioTek Instruments, Inc., Vermont, USA). The endpoint titres were calculated as the reciprocal of the last dilution reaching a cut-off value set to twice the mean optical density of a negative control [34,35].

### Statistics

Statistical differences were assessed using the Mann-Whitney U test or the Kruskal-Wallis test with Dunn’s post-hoc test calculated by GraphPad Prism 7 (GraphPad Software, San Diego, California, USA). *P* values lower than 0.05 were considered significant.

## Results

### Nasal immunisation with an antigen in combination with various polyphenols induces antigen-specific antibody responses

**Fig 2:**
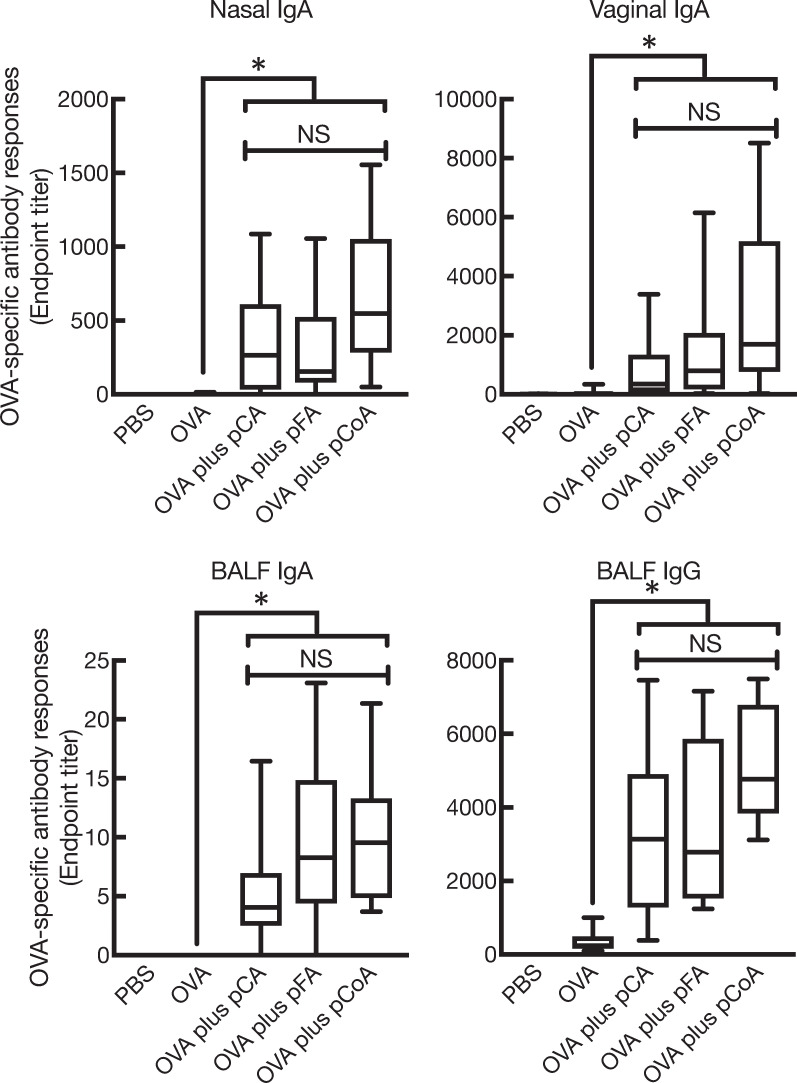
Enzymatically polymerised polyphenols promote antigen-specific mucosal antibody responses. Female BALB/cCrSlc mice were immunised three times intranasally (days 0, 7, and 14) with vehicle (PBS), OVA (2.5 μg/mouse) alone, OVA (2.5 μg/mouse) plus pCA (100 μg/mouse), OVA (2.5 μg/mouse) plus pFA (100 μg/mouse), or OVA (2.5 μg/mouse) plus pCoA (100 μg/mouse). OVA-specific IgA endpoint titres in nasal wash, BALF, and vaginal wash as well as OVA-specific IgG endpoint titres in BALF at day 21 were detected by ELISA. The data were obtained from three biologically independent experiments. PBS, *n* = 9; OVA, *n* = 9; OVA plus pCA, *n* = 9; OVA plus pFA, *n* = 9; OVA plus pCoA, *n* = 9. The box-plot shows the median value with the 25th–75th percentiles and the error bars indicate the 5th–95th percentiles. Significance was calculated using the Kruskal-Wallis with Dunn’s post-hoc test: *p < 0.05.

**Fig 3:**
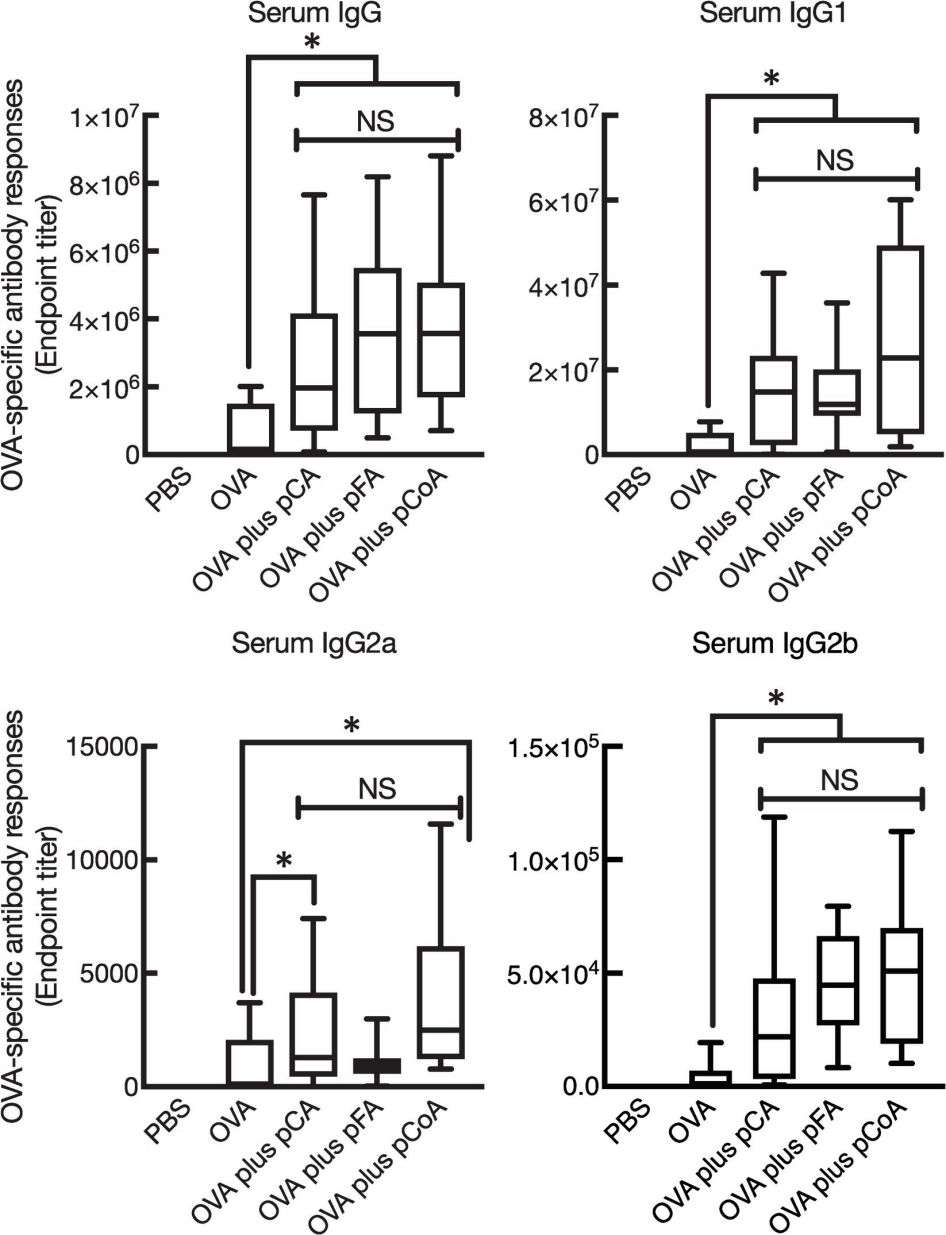
Enzymatically polymerised polyphenols promote antigen-specific systemic antibody responses. Female BALB/cCrSlc mice were immunised three times intranasally (days 0, 7, and 14) with vehicle (PBS), OVA (2.5 μg/mouse) alone, OVA (2.5 μg/mouse) plus pCA (100 μg/mouse), OVA (2.5 μg/mouse) plus pFA (100 μg/mouse), or OVA (2.5 μg/mouse) plus pCoA (100 μg/mouse). OVA-specific total IgG, IgG1, IgG2a, and IgG2b endpoint titres in sera at day 21 were detected by ELISA. The data were obtained from three biologically independent experiments. PBS, *n* = 9; OVA, *n* = 9; OVA plus pCA, *n* = 9; OVA plus pFA, *n* = 9; OVA plus pCoA, *n* = 9. The box-plot shows the median value with the 25th–75th percentiles and the error bars indicate the 5th–95th percentiles. Significance was calculated using the Kruskal-Wallis with Dunn’s post-hoc test: *p < 0.05.

To compare the influence of different sources of phenolic compounds on the promotion of antigen-specific antibody responses in both mucosal and systemic compartments, mucosal immunisation of the enzymatically polymerised polyphenols in combination with OVA in mice was performed intranasally. The first experiment was aimed to test whether all polyphenols synthesised in this study induced OVA-specific mucosal IgA and IgG responses. As expected, the mice with OVA plus pCA showed high levels of OVA-specific mucosal IgA and BALF IgG, as reported earlier (Fig 2). We next examined the mucosal adjuvant effects of pFA and pCoA. OVA plus pFA or pCoA also induced high levels of OVA-specific mucosal IgA as well as BALF IgG compared with mice immunised with OVA alone; the levels were similar to those generated by pCA (Fig 2). In addition to mucosal responses, pFA and pCoA also promoted OVA-specific systemic IgG in sera compared with mice immunised with OVA alone; the levels were comparable to those generated by pCA (Fig 3). On the contrary, OVA-specific IgE production in sera was not detected in any of the groups. Furthermore, OVA-specific IgM responses in sera were not altered when OVA was nasally co-administered with polyphenols compared to mice that received OVA alone (data not shown).

We further tested the production of OVA-specific IgG1, IgG2a, and IgG2b in sera from mice with OVA plus the polyphenols to assess the type of immune response induced. Intranasal immunisation with OVA plus all enzymatically polymerised polyphenols tested in this study exerted primarily OVA-specific IgG1 production rather than IgG2a and IgG2b antibodies (Fig 3). This production pattern implied an alteration to the Th2-type immune response since serum IgG subclasses reflect Th responses [36,37]. On the other hand, intranasal immunisation with OVA plus all enzymatically polymerised polyphenols did not induce OVA-specific serum IgM or IgE. Collectively, these data indicate that the enzymatically polymerised polyphenols possess mucosal adjuvant activities, which is not different from the precursors synthesised.

### Comparison of the mucosal adjuvant effect of polyphenols with a well-known experimental mucosal adjuvant, CT

**Fig 4:**
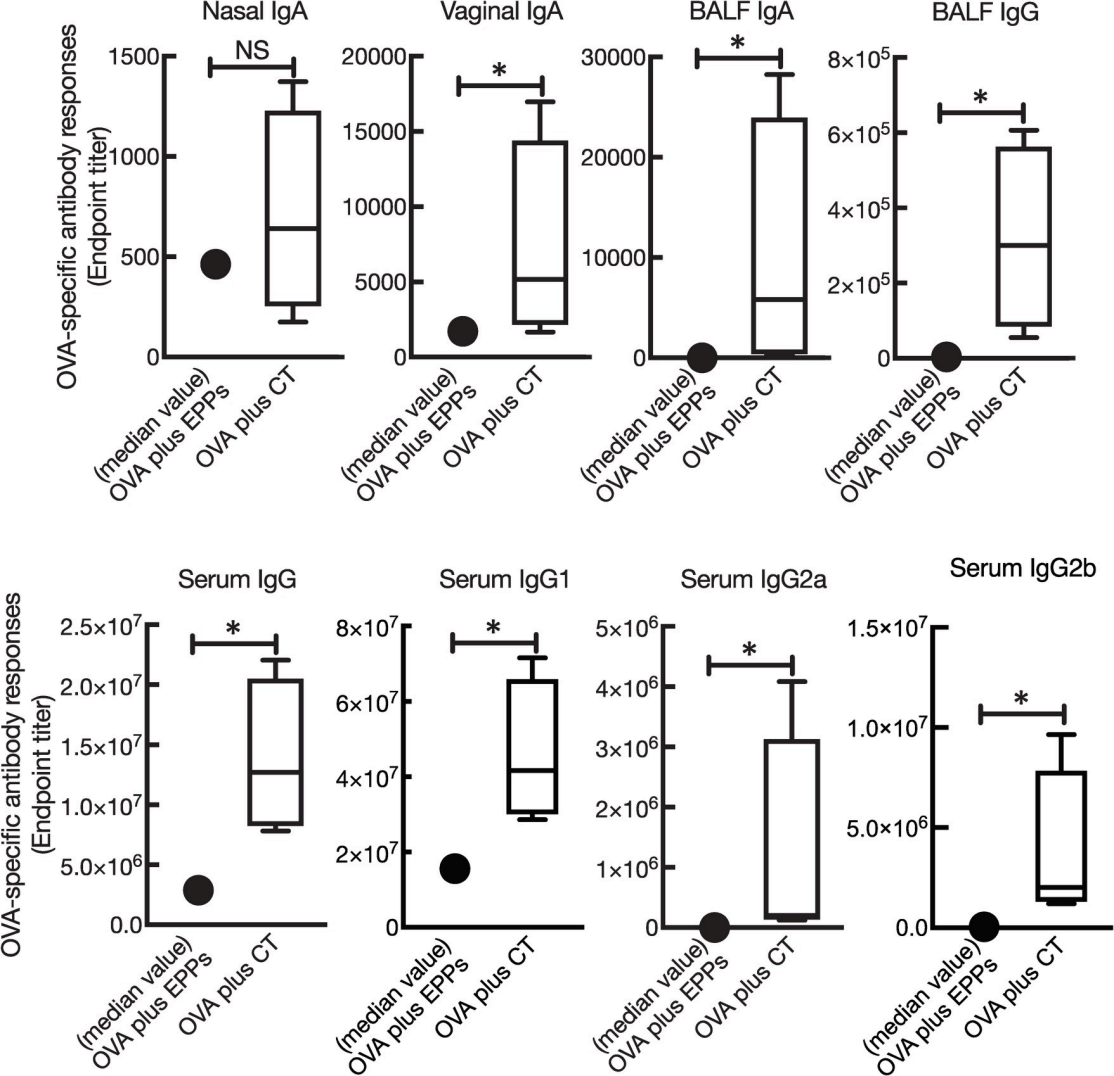
Comparison of the mucosal adjuvant effects of the enzymatically polymerised polyphenols with CT. Female BALB/cCrSlc mice were immunised three times intranasally (days 0, 7, and 14) with OVA (2.5 μg/mouse) plus either pCA (100 μg/mouse), pFA (100 μg/mouse), or pCoA (100 μg/mouse), or OVA (2.5 μg/mouse) plus CT (1 μg/mouse). ELISA detected OVA-specific IgA endpoint titres in nasal wash and BALF; IgG endpoint titres in BALF and vaginal wash; and total IgG, IgG1, and IgG2a endpoint titres in sera on day 21. The data were obtained from three biologically independent experiments. OVA plus enzymatically polymerised polyphenols (EPPs); the median value of the sum of OVA plus pCA, OVA plus pFA, and OVA plus pCoA, *n* = 27; OVA plus CT, *n* = 9. The box-plot shows the median value with the 25th–75th percentiles and the error bars indicate the 5th–95th percentiles. Significance was calculated using the Mann-Whitney U test: *p < 0.05.

In order to examine the efficacy of mucosal adjuvant activities of enzymatically polymerised polyphenols from various phenolic precursors, we next compared the mucosal adjuvant effects of polyphenols with CT, which is a potent mucosal adjuvant. Comparing the mucosal adjuvant effects of the enzymatically polymerised polyphenols (EPPs) and CT, Fig 4 shows that the mucosal adjuvant effects of the enzymatically polymerised polyphenols were relatively low in terms of nasal IgA, vaginal IgA, BALF IgA, and BALF IgG. In addition, the production of OVA-specific systemic IgG antibodies was relatively low.

## Discussion

Although mucosal vaccination is now considered as the most promising strategy in the prevention and/or treatment of fatal infectious diseases, no subunit mucosal vaccine is available to date. One of the major reasons is the lack of agents/methodologies for inducing antigen-specific immune responses in the mucosa. In this context, the development of agents/methodologies to exert the mucosal immune response that is safe and effective is needed. In the course of such studies, we have previously found that an enzymatically polymerised polyphenol from caffeic acid as a precursor can enhance antigen-specific immune responses when administered nasally with an antigen [32]. Nonetheless, we do not know if these effects rely upon the structure of a phenolic compound used as a precursor. In this study, we show that intranasal immunisation of all the tested polymers, enzymatically synthesised from various phenylpropanoids as precursors, potentiate the production of antigen-specific antibodies in both mucosal and systemic fluids. The results shown here may support the development of a safe and effective nasal vaccine system for preventing and/or treating infectious diseases.

Herein, we found that intranasal immunisation of pFA and pCoA in combination with OVA induced OVA-specific antibody responses similar to those of pCA (Figs 2 and 3) [32]. Moreover, the polyphenols preferentially induced Th2 responses based on the observation of higher antigen-specific IgG1 production, rather than the IgG2 response. Notably, a limitation of this study is that the production of cytokines in leukocytes from mice vaccinated with OVA in combination with polyphenols when restimulated with OVA *in vitro* has not been evaluated to further assess the type of immune response evoked by the polyphenols. The mucosal adjuvant activities of pFA and pCoA for the induction of antigen-specific mucosal and systemic antibody responses were not statistically significant compared to those induced by pCA (Figs 2 and 3). However, the immune-stimulatory effects of pFA and pCoA in terms of the production of cytokines by splenocytes stimulated with these polyphenols [e.g. IFN-γ, granulocyte macrophage colony-stimulating factor, and tumour necrosis factor-α] and by bone marrow dendritic cells stimulated with these polyphenols were higher than those of pCA [28–31]. A comparative study of the mucosal adjuvant effects of polyphenols on CT showed that the mucosal adjuvant effects of polyphenols were relatively weak (Fig 4). Nevertheless, polyphenols developed in this study could be a useful formulation for nasal vaccines because CT is a molecule derived from pathogenic *Vibrio cholerae*, the causative agent of diarrhoeal disease. However, despite recent significant efforts made to develop non-toxic mutants of CT, none of these have been approved for human use [38,39].

Although the mechanism of mucosal adjuvant effects of polyphenols has not been elucidated at this time, in addition to the immune-stimulatory activities of polyphenols, recent studies have revealed that polyphenols, such as epigallocatechin gallate (EGCG), can interact with proteins/peptides, which have the amino acid residues of proline, phenylalanine, and arginine, mainly through non-covalent hydrophobic or hydrophilic interactions, leading to complex formation [40,41]. Tachibana et al. have reported that EGCG could bind to the 67 kDa laminin receptor on the cell surface of various cell types, including DCs [42–44]. From these observations, we speculate that polyphenols also serve as an antigen delivery vehicle to mucosal DCs, resulting in subsequent antigen-specific immune responses. This possibility needs to be clarified in future experiments.

## Conclusions

In conclusion, we have elucidated that the mucosal adjuvant effects of enzymatically polymerised polyphenols did not rely upon the starting phenolic compounds as precursors. These biomaterials can be synthesised using food containing components such as caffeic acid and HRP as an enzymatic source. We believe that the enzymatically polymerised polyphenols could be used as a highly safe and efficient formulation for mucosal vaccine systems; thus, these biomaterials can be clinically utilised for combatting infectious diseases caused by deadly pathogens.
